# Supplementary material for: HIVprotI: an integrated web based platform for prediction and design of HIV proteins inhibitors
Source: J Cheminform. 2018 Mar 9;10:12. doi: 10.1186/s13321-018-0266-y (PMC5845081; doi:10.1186/s13321-018-0266-y)
Supplement: Supplementary file 2 — Additional file 2. Source code of HIVProtI web server. [file 13321_2018_266_MOESM2_ESM.zip › HIVprotI_Source-code/hivprotI/weka-3-6-6/doc/weka/experiment/ClassifierSplitEvaluator.html]

ClassifierSplitEvaluator


---


|  |  |  |
| --- | --- | --- |
| **Overview** \| **Package** \| **Class** \| **Tree** \| **Deprecated** \| **Index** \| **Help** \| **Weka's home** | |  |
| **PREV CLASS**   **NEXT CLASS** | **FRAMES**    **NO FRAMES**     **All Classes** |  |
| SUMMARY: NESTED \| FIELD \| CONSTR \| METHOD | DETAIL: FIELD \| CONSTR \| METHOD |  |


---


## weka.experiment Class ClassifierSplitEvaluator

```
java.lang.Object
  weka.experiment.ClassifierSplitEvaluator
```

**All Implemented Interfaces:**: java.io.Serializable, AdditionalMeasureProducer, OptionHandler, RevisionHandler, SplitEvaluator

**Direct Known Subclasses:**: CostSensitiveClassifierSplitEvaluator

---

``` public class ClassifierSplitEvaluator extends java.lang.Object implements SplitEvaluator, OptionHandler, AdditionalMeasureProducer, RevisionHandler ```

A SplitEvaluator that produces results for a classification scheme on a nominal class attribute.


Valid options are:

```
 -W <class name>
  The full class name of the classifier.
  eg: weka.classifiers.bayes.NaiveBayes
```

```
 -C <index>
  The index of the class for which IR statistics
  are to be output. (default 1)
```

```
 -I <index>
  The index of an attribute to output in the
  results. This attribute should identify an
  instance in order to know which instances are
  in the test set of a cross validation. if 0
  no output (default 0).
```

```
 -P
  Add target and prediction columns to the result
  for each fold.
```

```
 Options specific to classifier weka.classifiers.rules.ZeroR:
```

```
 -D
  If set, classifier is run in debug mode and
  may output additional info to the console
```

All options after -- will be passed to the classifier.

**Version:**
:   $Revision: 7513 $

**Author:**
:   Len Trigg (trigg@cs.waikato.ac.nz)

**See Also:**: Serialized Form

---

**Constructor Summary**

`ClassifierSplitEvaluator()`
  
          No args constructor.


| **Method Summary** | |
| --- | --- |
| `java.lang.String` | `classifierTipText()`             Returns the tip text for this property |
| `java.util.Enumeration` | `enumerateMeasures()`             Returns an enumeration of any additional measure names that might be in the classifier |
| `int` | `getAttributeID()`             Get the index of Attibute Identifying the instances |
| `int` | `getClassForIRStatistics()`             Get the value of ClassForIRStatistics. |
| `Classifier` | `getClassifier()`             Get the value of Classifier. |
| `java.lang.Object[]` | `getKey()`             Gets the key describing the current SplitEvaluator. |
| `java.lang.String[]` | `getKeyNames()`             Gets the names of each of the key columns produced for a single run. |
| `java.lang.Object[]` | `getKeyTypes()`             Gets the data types of each of the key columns produced for a single run. |
| `double` | `getMeasure(java.lang.String additionalMeasureName)`             Returns the value of the named measure |
| `java.lang.String[]` | `getOptions()`             Gets the current settings of the Classifier. |
| `boolean` | `getPredTargetColumn()` |
| `java.lang.String` | `getRawResultOutput()`             Gets the raw output from the classifier |
| `java.lang.Object[]` | `getResult(Instances train, Instances test)`             Gets the results for the supplied train and test datasets. |
| `java.lang.String[]` | `getResultNames()`             Gets the names of each of the result columns produced for a single run. |
| `java.lang.Object[]` | `getResultTypes()`             Gets the data types of each of the result columns produced for a single run. |
| `java.lang.String` | `getRevision()`             Returns the revision string. |
| `java.lang.String` | `globalInfo()`             Returns a string describing this split evaluator |
| `java.util.Enumeration` | `listOptions()`             Returns an enumeration describing the available options.. |
| `void` | `setAdditionalMeasures(java.lang.String[] additionalMeasures)`             Set a list of method names for additional measures to look for in Classifiers. |
| `void` | `setAttributeID(int v)`             Set the index of Attibute Identifying the instances |
| `void` | `setClassForIRStatistics(int v)`             Set the value of ClassForIRStatistics. |
| `void` | `setClassifier(Classifier newClassifier)`             Sets the classifier. |
| `void` | `setClassifierName(java.lang.String newClassifierName)`             Set the Classifier to use, given it's class name. |
| `void` | `setOptions(java.lang.String[] options)`             Parses a given list of options. |
| `void` | `setPredTargetColumn(boolean v)`             Set the flag for prediction and target output. |
| `java.lang.String` | `toString()`             Returns a text description of the split evaluator. |

**Methods inherited from class java.lang.Object**

`equals, getClass, hashCode, notify, notifyAll, wait, wait, wait`

**Constructor Detail**

### ClassifierSplitEvaluator

```
public ClassifierSplitEvaluator()
```

:   No args constructor.


**Method Detail**

### globalInfo

```
public java.lang.String globalInfo()
```

:   Returns a string describing this split evaluator

    :   **Returns:**: a description of the split evaluator suitable for displaying in the explorer/experimenter gui

---


### listOptions

```
public java.util.Enumeration listOptions()
```

:   Returns an enumeration describing the available options..

    :   **Specified by:**: `listOptions` in interface `OptionHandler`
    :   **Returns:**: an enumeration of all the available options.

---


### setOptions

```
public void setOptions(java.lang.String[] options)
                throws java.lang.Exception
```

:   Parses a given list of options.


    Valid options are:

    ```
     -W <class name>
      The full class name of the classifier.
      eg: weka.classifiers.bayes.NaiveBayes
    ```

    ```
     -C <index>
      The index of the class for which IR statistics
      are to be output. (default 1)
    ```

    ```
     -I <index>
      The index of an attribute to output in the
      results. This attribute should identify an
      instance in order to know which instances are
      in the test set of a cross validation. if 0
      no output (default 0).
    ```

    ```
     -P
      Add target and prediction columns to the result
      for each fold.
    ```

    ```
     Options specific to classifier weka.classifiers.rules.ZeroR:
    ```

    ```
     -D
      If set, classifier is run in debug mode and
      may output additional info to the console
    ```

    All options after -- will be passed to the classifier.

    :   **Specified by:**: `setOptions` in interface `OptionHandler`
    :   **Parameters:**: `options` - the list of options as an array of strings **Throws:**: `java.lang.Exception` - if an option is not supported

---


### getOptions

```
public java.lang.String[] getOptions()
```

:   Gets the current settings of the Classifier.

    :   **Specified by:**: `getOptions` in interface `OptionHandler`
    :   **Returns:**: an array of strings suitable for passing to setOptions

---


### setAdditionalMeasures

```
public void setAdditionalMeasures(java.lang.String[] additionalMeasures)
```

:   Set a list of method names for additional measures to look for
    in Classifiers. This could contain many measures (of which only a
    subset may be produceable by the current Classifier) if an experiment
    is the type that iterates over a set of properties.

    :   **Specified by:**: `setAdditionalMeasures` in interface `SplitEvaluator`
    :   **Parameters:**: `additionalMeasures` - a list of method names

---


### enumerateMeasures

```
public java.util.Enumeration enumerateMeasures()
```

:   Returns an enumeration of any additional measure names that might be
    in the classifier

    :   **Specified by:**: `enumerateMeasures` in interface `AdditionalMeasureProducer`
    :   **Returns:**: an enumeration of the measure names

---


### getMeasure

```
public double getMeasure(java.lang.String additionalMeasureName)
```

:   Returns the value of the named measure

    :   **Specified by:**: `getMeasure` in interface `AdditionalMeasureProducer`
    :   **Parameters:**: `additionalMeasureName` - the name of the measure to query for its value **Returns:**: the value of the named measure **Throws:**: `java.lang.IllegalArgumentException` - if the named measure is not supported

---


### getKeyTypes

```
public java.lang.Object[] getKeyTypes()
```

:   Gets the data types of each of the key columns produced for a single run.
    The number of key fields must be constant
    for a given SplitEvaluator.

    :   **Specified by:**: `getKeyTypes` in interface `SplitEvaluator`
    :   **Returns:**: an array containing objects of the type of each key column. The objects should be Strings, or Doubles.

---


### getKeyNames

```
public java.lang.String[] getKeyNames()
```

:   Gets the names of each of the key columns produced for a single run.
    The number of key fields must be constant
    for a given SplitEvaluator.

    :   **Specified by:**: `getKeyNames` in interface `SplitEvaluator`
    :   **Returns:**: an array containing the name of each key column

---


### getKey

```
public java.lang.Object[] getKey()
```

:   Gets the key describing the current SplitEvaluator. For example
    This may contain the name of the classifier used for classifier
    predictive evaluation. The number of key fields must be constant
    for a given SplitEvaluator.

    :   **Specified by:**: `getKey` in interface `SplitEvaluator`
    :   **Returns:**: an array of objects containing the key.

---


### getResultTypes

```
public java.lang.Object[] getResultTypes()
```

:   Gets the data types of each of the result columns produced for a
    single run. The number of result fields must be constant
    for a given SplitEvaluator.

    :   **Specified by:**: `getResultTypes` in interface `SplitEvaluator`
    :   **Returns:**: an array containing objects of the type of each result column. The objects should be Strings, or Doubles.

---


### getResultNames

```
public java.lang.String[] getResultNames()
```

:   Gets the names of each of the result columns produced for a single run.
    The number of result fields must be constant
    for a given SplitEvaluator.

    :   **Specified by:**: `getResultNames` in interface `SplitEvaluator`
    :   **Returns:**: an array containing the name of each result column

---


### getResult

```
public java.lang.Object[] getResult(Instances train,
                                    Instances test)
                             throws java.lang.Exception
```

:   Gets the results for the supplied train and test datasets. Now performs
    a deep copy of the classifier before it is built and evaluated (just in case
    the classifier is not initialized properly in buildClassifier()).

    :   **Specified by:**: `getResult` in interface `SplitEvaluator`
    :   **Parameters:**: `train` - the training Instances.: `test` - the testing Instances. **Returns:**: the results stored in an array. The objects stored in the array may be Strings, Doubles, or null (for the missing value). **Throws:**: `java.lang.Exception` - if a problem occurs while getting the results

---


### classifierTipText

```
public java.lang.String classifierTipText()
```

:   Returns the tip text for this property

    :   **Returns:**: tip text for this property suitable for displaying in the explorer/experimenter gui

---


### getClassifier

```
public Classifier getClassifier()
```

:   Get the value of Classifier.

    :   **Returns:**: Value of Classifier.

---


### setClassifier

```
public void setClassifier(Classifier newClassifier)
```

:   Sets the classifier.

    :   **Parameters:**: `newClassifier` - the new classifier to use.

---


### getClassForIRStatistics

```
public int getClassForIRStatistics()
```

:   Get the value of ClassForIRStatistics.

    :   **Returns:**: Value of ClassForIRStatistics.

---


### setClassForIRStatistics

```
public void setClassForIRStatistics(int v)
```

:   Set the value of ClassForIRStatistics.

    :   **Parameters:**: `v` - Value to assign to ClassForIRStatistics.

---


### getAttributeID

```
public int getAttributeID()
```

:   Get the index of Attibute Identifying the instances

    :   **Returns:**: index of outputed Attribute.

---


### setAttributeID

```
public void setAttributeID(int v)
```

:   Set the index of Attibute Identifying the instances

    :   **Parameters:**: `v` - index the attribute to output

---


### getPredTargetColumn

```
public boolean getPredTargetColumn()
```

:   **Returns:**: true if the prediction and target columns must be outputed.

---


### setPredTargetColumn

```
public void setPredTargetColumn(boolean v)
```

:   Set the flag for prediction and target output.

    :   **Parameters:**: `v` - true if the 2 columns have to be outputed. false otherwise.

---


### setClassifierName

```
public void setClassifierName(java.lang.String newClassifierName)
                       throws java.lang.Exception
```

:   Set the Classifier to use, given it's class name. A new classifier will be
    instantiated.

    :   **Parameters:**: `newClassifierName` - the Classifier class name. **Throws:**: `java.lang.Exception` - if the class name is invalid.

---


### getRawResultOutput

```
public java.lang.String getRawResultOutput()
```

:   Gets the raw output from the classifier

    :   **Specified by:**: `getRawResultOutput` in interface `SplitEvaluator`
    :   **Returns:**: the raw output from th,0e classifier

---


### toString

```
public java.lang.String toString()
```

:   Returns a text description of the split evaluator.

    :   **Overrides:**: `toString` in class `java.lang.Object`
    :   **Returns:**: a text description of the split evaluator.

---


### getRevision

```
public java.lang.String getRevision()
```

:   Returns the revision string.

    :   **Specified by:**: `getRevision` in interface `RevisionHandler`
    :   **Returns:**: the revision


---


|  |  |  |
| --- | --- | --- |
| **Overview** \| **Package** \| **Class** \| **Tree** \| **Deprecated** \| **Index** \| **Help** \| **Weka's home** | |  |
| **PREV CLASS**   **NEXT CLASS** | **FRAMES**    **NO FRAMES**     **All Classes** |  |
| SUMMARY: NESTED \| FIELD \| CONSTR \| METHOD | DETAIL: FIELD \| CONSTR \| METHOD |  |


---
